# Retinal degeneration in *rpgra* mutant zebrafish

**DOI:** 10.3389/fcell.2023.1169941

**Published:** 2023-06-07

**Authors:** Xiliang Liu, Shanshan Han, Fei Liu, Shanshan Yu, Yayun Qin, Jingzhen Li, Danna Jia, Pan Gao, Xiang Chen, Zhaohui Tang, Mugen Liu, Yuwen Huang

**Affiliations:** ^1^ Key Laboratory of Molecular Biophysics of Ministry of Education, Department of Genetics and Developmental Biology, College of Life Science and Technology, Huazhong University of Science and Technology, Wuhan, Hubei, China; ^2^ Sansure Biotech Inc., Changsha, Hunan, China; ^3^ Medical College, China Three Gorges University, Yichang, China; ^4^ The Institute of Infection and Inflammation, China Three Gorges University, Yichang, Hubei, China; ^5^ State Key Laboratory of Freshwater Ecology and Biotechnology, Institute of Hydrobiology, Innovation Academy for Seed Design, Chinese Academy of Science, Wuhan, Hubei, China; ^6^ Institute of Visual Neuroscience and Stem Cell Engineering, College of Life Sciences and Health, Wuhan University of Science and Technology, Wuhan, Hubei, China; ^7^ Maternal and Child Health Hospital of Hubei Province, Tongji Medical College, Huazhong University of Science and Technology, Wuhan, Hubei, China

**Keywords:** *rpgra*, zebrafish, TALEN, ciliary transport, RAB8A, retinal degeneration

## Abstract

**Introduction:** Pathogenic mutations in *RPGR* ^*ORF15*^, one of two major human *RPGR* isoforms, were responsible for most X-linked retinitis pigmentosa cases. Previous studies have shown that *RPGR* plays a critical role in ciliary protein transport. However, the precise mechanisms of disease triggered by *RPGR* ^*ORF15*^ mutations have yet to be clearly defined. There are two homologous genes in zebrafish, *rpgra* and *rpgrb*. Zebrafish *rpgra* has a single transcript homologous to human *RPGR* ^*ORF15*^; *rpgrb* has two major transcripts: *rpgrb* ^*ex1-17*^ and *rpgrb* ^*ORF15*^, similar to human *RPGR* ^*ex1-19*^ and *RPGR* ^*ORF15*^, respectively. *rpgrb* knockdown in zebrafish resulted in both abnormal development and increased cell death in the dysplastic retina. However, the impact of knocking down *rpgra* in zebrafish remains undetermined. Here, we constructed a *rpgra* mutant zebrafish model to investigate the retina defect and related molecular mechanism.

**Methods:** we utilized transcription activator-like effector nuclease (TALEN) to generate a *rpgra* mutant zebrafish. Western blot was used to determine protein expression. RT-PCR was used to quantify gene transcription levels. The visual function of embryonic zebrafish was detected by electroretinography. Immunohistochemistry was used to observe the pathological changes in the retina of mutant zebrafish and transmission electron microscope was employed to view subcellular structure of photoreceptor cells.

**Results:** A homozygous *rpgra* mutant zebrafish with c.1675_1678delins21 mutation was successfully constructed. Despite the normal morphological development of the retina at 5 days post-fertilization, visual dysfunction was observed in the mutant zebrafish. Further histological and immunofluorescence assays indicated that *rpgra* mutant zebrafish retina photoreceptors progressively began to degenerate at 3-6 months. Additionally, the mislocalization of cone outer segment proteins (Opn1lw and Gnb3) and the accumulation of vacuole-like structures around the connecting cilium below the OSs were observed in mutant zebrafish. Furthermore, Rab8a, a key regulator of opsin-carrier vesicle trafficking, exhibited decreased expression and evident mislocalization in mutant zebrafish.

**Discussion:** This study generated a novel *rpgra* mutant zebrafish model, which showed retinal degeneration. our data suggested Rpgra is necessary for the ciliary transport of cone-associated proteins, and further investigation is required to determine its function in rods. The *rpgra* mutant zebrafish constructed in this study may help us gain a better understanding of the molecular mechanism of retinal degeneration caused by *RPGR* ^*ORF15*^ mutation and find some useful treatment in the future.

## 1 Introduction

Hereditary retinitis pigmentosa (RP) is a condition leading to photoreceptor degeneration that affects approximately 1/3,000 to 1/7,000 people worldwide, commonly resulting in severe visual loss and, eventually, blindness (O'Neal and Luther. 2022; Wright et al., 2010). It is a genetically and clinically heterogeneous progressive disease of the retina (Sahel et al., 2014). X-linked RP (XLRP) is one of the most severe forms. Approximately 70%–90% of XLRP cases are caused by mutations in the RPGR gene, with RPGR mutations accounting for 10%–15% of all RP cases (Gill et al., 2019). RPGR mutations are also associated with other retinal dystrophies, such as cone-rod dystrophy and atrophic macular degeneration, indicating that RPGR is crucial for the maintenance of retinal stability (Ayyagari et al., 2002; Wang et al., 2021).

The human *RPGR* has two major transcripts, *RPGR* ^*EX1-19*^ and *RPGR* ^*ORF15*^ (Meindl et al., 1996; Vervoort et al., 2000). They share exons 1 to 14 that contain an RCC1-like domain, and the ORF15 exon has a repetitive region in glycine and glutamic acid which is a mutation hot spot (Shu et al., 2008; Vervoort et al., 2000). *RPGR* ^*ORF15*^ encodes an 1,152 amino acid protein that is most strongly expressed in the retina and is localized to the connecting cilium of the photoreceptor (CC) (Hong et al., 2000; Hong et al., 2003; Mavlyutov et al., 2002). Biochemical studies and various *postmortem* studies using immunohistochemical techniques suggest that RPGR^ORF15^ plays a role in ciliary transportation (Adamian et al., 2006; Beltran et al., 2009, 2006; Hong et al., 2000; Khanna et al., 2005; Thompson et al., 2012).

Various animal models have been utilized to study the function of RPGR. Two dog models (XLPRA1 and XLPRA2) with different mutations in exon ORF15 of the *RPGR* gene were described in 2002 (Zhang et al., 2002). The XLPRA1 mutant dog (five base deletions resulting in a frameshift and immediate premature stop) had a normal retina function until 6 months, followed by a retina degeneration that first involved rods. The dog with XLPRA2 mutation that caused a long frameshift with 34 additional basic residues had a more severe degeneration with abnormal retinal development (Beltran et al., 2012, 2006). To date, several mouse models have been characterized, including one carrying a deletion of exons four to six in the *Rpgr* gene. It demonstrated slow degeneration with initial opsin mislocalization followed by decreased rhodopsin protein level (Hong et al., 2000). Another mouse model with a 5bp deletion in exon 8 also displayed slow but progressive age-related retinal degeneration (Hu et al., 2020), while the *Rpgr* exon1 conditional knockout mice demonstrated faster retinal degeneration compared to *Rpgr*-KO mice (Huang et al., 2012). A naturally occurring 32bp deletion in *Rpgr* ^*ORF15*^ in rd9 mice caused much slower degeneration with features resembling XLRP with mutations in *RPGR* exon ORF15 (Falasconi et al., 2019).

The zebrafish model has now become a valuable instrument in the investigation of human eye diseases (Lieschke and Currie, 2007; Raghupathy et al., 2013). Two zebrafish genes, *rpgra* and *rpgrb*, have been identified as homologous to human *RPGR*. *rpgra* is located on chromosome 9 and contains 13 exons that encode a protein of 1,698 amino acids. *rpgrb* is located on chromosome 11 and has two transcripts: one is *rpgrb* ^*ORF15*^ consisting of 14 exons encoding 1,413 amino acids, and the other one is *rpgrb* ^*ex1-17*^, encoding 708 amino acids with 17 exons (Shu et al., 2010). Comparing genes up and downstream of *rpgr* between zebrafish, *Fugu, Xenopus*, lizard, chicken, and humans, *rpgra* shared syntenic genes with mammals, while *rpgrb* shows the same syntenic relationships as the Fugu (Raghupathy et al., 2015). Bioinformatic alignments revealed Rpgra and Rpgrb^ORF15^ are homologous to human RPGR^ORF15^, of which Rpgra displays greater amino acid identity with human RPGR in the ORF15 domain, and Rpgrb^ORF15^ displays greater identity in the RCCL domain (Shu et al., 2010). Previous studies showed that knockdown of *rpgrb* ^*ORF15*^ in zebrafish resulted in reduced length of Kupffer’s vesicle (KV) cilia and is associated with ciliary anomalies including shortened body-axis, kinked tail, hydrocephaly, and edema but does not affect retinal development (Ghosh et al., 2010). Moreover, the simultaneous knockdown of *rpgrb* ^*ORF15*^ and *rpgrb* ^*ex1-19*^ expression led to developmental defects, affecting gastrulation, tail, head, and eye development. Developmental abnormalities in the eye included lamination defects, failure to develop photoreceptor outer segments, and a small eye phenotype, associated with increased cell death throughout the retina, while the inhibition of *rpgra* expression was not detected as a significant defect (Shu et al., 2010). However, Gerner et al. found that morpholino knock-down of *rpgra* ^*ORF15*^ caused developmental defects including abnormal body curvature, cerebral abnormalities, underdeveloped eyes, and pronephric cysts (Gerner et al., 2010). To ascertain the function of *rpgra* in the zebrafish eyes, investigate the pathogenic process, and obtain a better understanding of the molecular mechanisms of disease caused by *RPGR* ^*ORF15*^ mutations, a stable defect model is urgently needed.

In our study, we constructed a *rpgra* mutant zebrafish model using a Transcription activator-like effector nuclease (TALEN) technology. In the mutant line, we found that early retinal function was affected; the length of the photoreceptor outer segment (OS) and the thickness of the outer nuclear layer (ONL) decreased progressively with age. Furthermore, the mislocalization of red opsin protein (Opn1lw), Gnb3, and Rab8a was observed along with the accumulation of abnormal vacuole-like structures in photoreceptors. These observations indicate a progressive retinal degeneration in *rpgra* mutant zebrafish and highlight the crucial role played by Rpgra in opsin proteins transportation, thereby enhancing our understanding of the function of Rpgra in zebrafish retina and *RPGR* ^*ORF15*^ mutant disease pathogenesis.

## 2 Materials and methods

### 2.1 Zebrafish maintenance

The AB strain of zebrafish was kept in a recirculating water system at 26°C–28.5°C under a 14-h light/10-h dark cycle. Embryos were kept in an E3 medium at 30°C. They were fed three times a day with fresh paramecia or brine shrimp. Control wild-type lines in this paper were derived from wild-type immature zygotic embryo culture.

### 2.2 TALEN construction and microinjection

The gene sequence information for zebrafish *rpgra* (ENSDART00000079095.4) was acquired from Ensembl (http://asia.ensembl.org/index.html) (Cunningham et al., 2022). We used online tools TAL Effector Nucleotide Targeter2.0 (https://tale-nt.cac.cornell.edu/) (Doyle et al., 2012) to design TALENs that target the exon13 of *rpgra*; the left target sequence was 5′ AAC​AGA​ATC​TCA​ATC​ATC​AA 3′ and the right was 5′ GCA​TCT​CCA​GGC​TGG​GCT​T 3′. The plasmids of the TALENs were assembled by using the Golden Gate TALEN kit according to the operating manual (Cermak et al., 2011); the 86 library vectors in this kit were available from *Addgene*, and TALEN mRNAs were *in vitro* transcribed and purified using T3 mMessage mMachine Kit (Ambion, Austin, TX, United States). A pair of left and right TALEN mRNAs was mixed at a ratio of 1:1; the final concentration of each arm was 100 ng/μL and it was then microinjected into one-cell stage egg yolks of wild-type zebrafish.

### 2.3 *rpgra* mutant zebrafish screening

**FIGURE 1:**
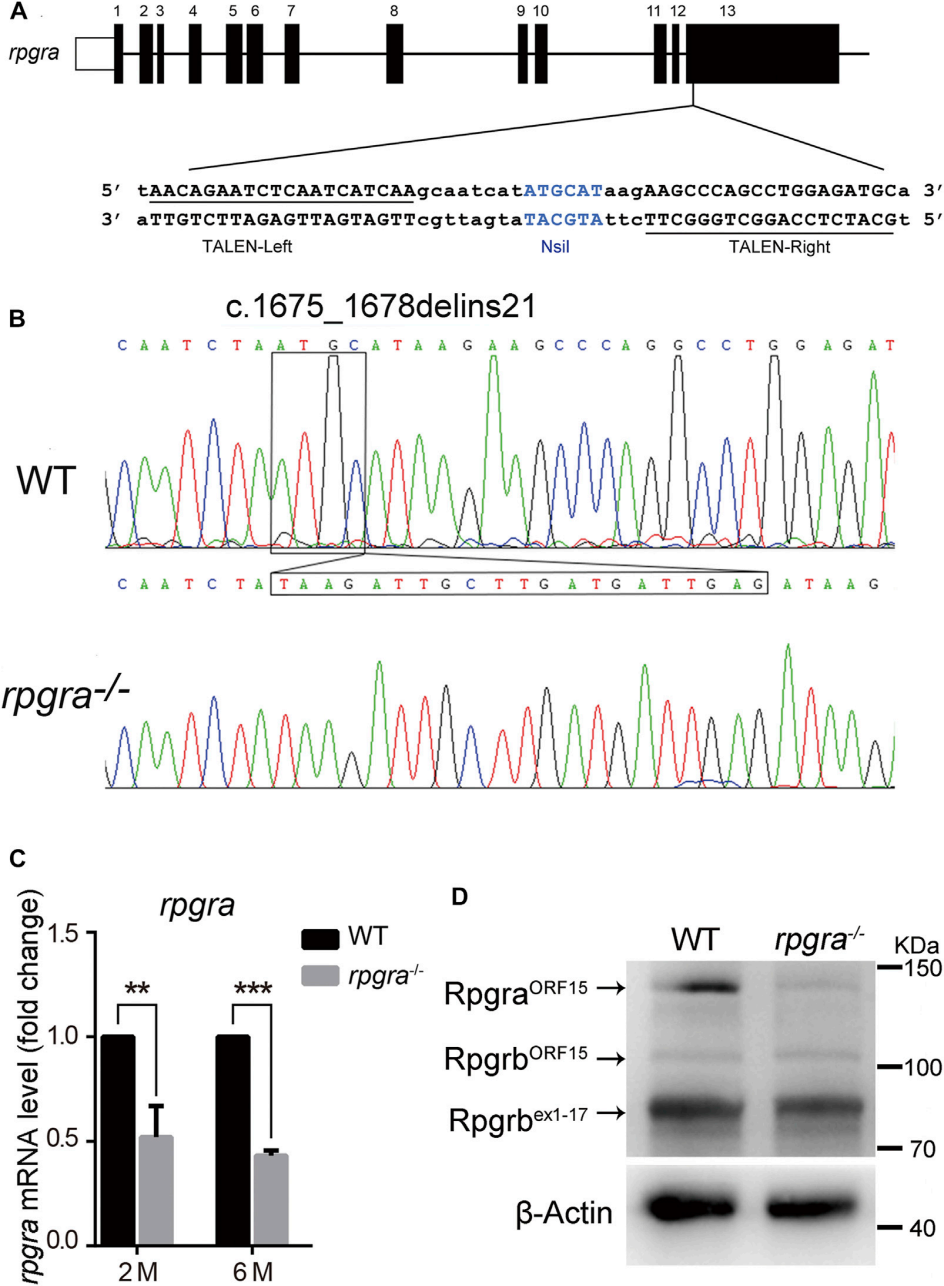
Generation of the *rpgra* mutant zebrafish. **(A)** The 13 exons of zebrafish *rpgra* are shown with the left and right arms of the TALEN binding sequences underlined. TALEN-L and TALEN-R, the left and right arms of the TALENs binding sequences; the NsiI restriction site in the spacer region is used for mutation detection. **(B)** Sequencing of the c.1675_1678delinsTAAGATTGCTTGATGATTGAG *rpgra* mutation in homozygous zebrafish. The DNA base change was indicated with a box. **(C)** *rpgra* mRNA levels in 2- and 6-month-old WT and *rpgra* ^*−/−*^ zebrafish eyes detected by quantitative PCR. A*ctb1* served as endogenous control. The result was shown as mean ± SD. **, 0.001 < *p* < 0.01; ***, *p* < 0.001. **(D)** Rpgra protein levels of WT and *rpgra* ^*−/−*^ zebrafish at 2mpf were revealed by Western blot using the anti-RPGR antibody.

Two days after injection, 10 embryos were collected from each dish of injected eggs, and genomic DNA was extracted. A 496bp DNA fragment containing the *rpgra* target site was amplified by PCR, using the flowing primers: Forward primer: ACG​TAT​TTC​AGC​AGG​CTC​TG and Reverse primer: GGA​GAT​TGG​ACC​TCT​TGA​GTG. The efficiency of the TALEN-mediated mutagenesis was determined by the restriction enzyme NsiI digestion analysis (Figure 1A). The rest of the embryos were raised to sexual maturity (3 months old) and outcrossed with wild-type zebrafish to obtain the first-generation heterozygous mutant zebrafish. The F1 zebrafish genotype also was determined by PCR and sequencing, then the same zebrafish genotype was selected to obtain the second generation, which contained the homozygote mutant zebrafish.

### 2.4 Electroretinography (ERG)

Protocols for the zebrafish larvae ERG recordings were described previously (Fleisch et al., 2008; Han et al., 2018; Lu et al., 2017). In brief, after 30 min of dark adaption, zebrafish larvae at 5 dpf were paralyzed with Esmeron (0.8 mg/mL in E3 medium; MedChem Express). The larvae were then placed on a wet filter paper over the reference electrode and the recording electrode was placed on the center of the cornea. ERGs were recorded after 5 min of complete dark adaption. A 1–2 s single stimulus with 6,000 lux illuminance was used to generate a typical ERG trace. All the traces were collected within 20 min, and the average of the top five b-wave amplitudes was regarded as the larva’s b-wave amplitude.

### 2.5 Histologic analysis

Zebrafish eyes were isolated and fixed with 4% paraformaldehyde (PFA) for 8–12 h at 4°C; 4% PFA was removed and rinsed in 1x PBS, and the specimen was soaked in 30% sucrose and dissolved in 1x PBS at room temperature until the eyes sank to the bottom of the tube, and the eyes were embedded in OCT compound (SAKURA Tissue-Tek^®^ OCT compound, United States). Embedded tissues were sliced along the vertical meridian of each eyeball (12 µm thick). Sections containing the whole retina were stained with hematoxylin and eosin (Beyotime, C0105S, China). For each section, digitized images of the retina were captured using Olympus-BX53. At least eight eyes from each genotype group were included in this analysis.

### 2.6 Immunofluorescence

For immunofluorescence staining, cryosections were rinsed with PDT (PBS solution containing 1% DMSO and 0.1% Triton X-100) for 10min and blocked with blocking solution (PDT containing 1% BSA and 10% normal goat serum) for 1 h at RT. Primary antibodies (Supplementary Table S1) were prepared in a blocking solution containing 2% normal goat serum and the slides were incubated overnight at 4°C. The slides were washed three times with PDT and incubated with Alexa Fluor 488 nm or 594 nm secondary antibodies (1:1,000; Molecular Probes^®^) for 1 h at 37°C. DAPI was diluted with PBS to a final 5 µg/mL and was used to label the nucleus. The slides were washed three times with PBS and then mounted under glass coverslips. Fluorescence images were captured using a confocal laser-scanning microscope (FluoViewTM FV1000 confocal microscope, Olympus Imaging).

### 2.7 Image analysis

For the analysis, we designated a reference region in the dorsal retina located 100 μm–200 μm away from the optic nerve (Figure 3A). This reference region was subsequently utilized for comparative analysis in various contexts. The quantification of the outer nuclear layer (ONL) thickness, photoreceptor layer (outer retinal) thickness, and outer segment length in Figures 3, 4, and Supplementary Figure S4 were performed by averaging measurements in our reference region from three sections chosen from each retina (eight retinas from four individual fish per group). All chosen sections had a visible optic nerve. The average thickness or length was assessed using the measurement tool in Photoshop; eight points from the reference region were used to measure and the results were averaged. The thickness of ONL was measured as the interface between the outer plexiform layer and the photoreceptor inner segment. The photoreceptor layer thickness was measured from the outer plexiform layer to the inner surface of the RPE layer. In addition, the length of the cones or rod outer segments depicted in Figure 4 was measured and averaged from 10 random photoreceptors. The cones within the reference region were manually counted based on the staining signals of a specific cone opsin.

### 2.8 TUNEL staining

TUNEL staining was performed using the TUNEL BrightRed Apoptosis Detection Kit (Vazyme Biotech) according to the manufacturer’s instructions. Generally, cryosections were air-dried at RT and then fixed with 4% paraformaldehyde in PBS for 30 min. The slides were washed two times with PBS for 15 min and incubated with the proteinase K buffer for 10 min. After that, the slides were washed 2–3 times with PBS and incubated with the equilibration buffer for 10–30 min. Then, the retinal sections were incubated in TdT buffer at 4°C overnight. The next day, following DAPI labeling, the slides were mounted under glass coverslips.

### 2.9 Transmission electron microscopy

Zebrafish eyes were isolated and left in the fixative (2.5% glutaraldehyde in 0.1 M PBS buffer, pH 7.4) overnight at 4°C. After they were fixed, the eyes were sent to Servicebio Company, and the subsequent operation was mainly completed by the company, briefly described as follows. After three washes with PBS, the eyes were further fixed in 1% osmium tetroxide for 2 h at room temperature (RT) and then dehydrated through an ethanol gradient, followed by treatment with propylene oxide and embedded in an epoxy medium. Embedded eyes were sliced into ultrathin sections (100 nm) using a Reichert-Jung ultramicrotome (Leica). Sections were stained with 3% uranyl acetate and 3% lead citrate for 15 min and visualized with a transmission electron microscope system (HT7700, Hitachi).

### 2.10 RT-PCR

The total RNA of zebrafish was extracted using TRIzol (Takara) and quantitated by NanoDrop spectrometry (Thermo Scientific, Wilmington, DE). The cDNA was generated by HiScript Q RT SuperMix (Vazyme). Realtime PCR was performed using AceQ^®^ qPCR SYBR^®^ Green Master Mix (Vazyme) according to the manufacturer’s instructions, and relative gene expression was quantified using the StepOnePlusTM Real-Time PCR System (Life Technologies). Gene primers are listed in Supplementary Table S2.

### 2.11 Western blot

Zebrafish eyes were isolated and homogenized in a cold RIPA lysis buffer with a protease inhibitor cocktail. Protein concentration was determined using the BCA protein assay kit (Beyotime, China). Proteins were separated on SDS-PAGE and transferred to nitrocellulose membranes. The membranes were blocked for 2 h at room temperature (RT) in 5% skimmed milk dissolved in TBST buffer, and then incubated with the dilution solution of primary antibodies (Supplementary Table S1) overnight at 4°C with gentle agitation. After washing in TBST buffer (20 mM Tris–HCl, 150 mM NaCl, 0.05% Tween 20, and pH 7.6), the membranes were incubated with HRP-conjugated secondary antibodies (1:20,000; Thermo) for 2 h at RT. The membranes were then developed using SuperSignal®ELISA Femto Maximum Sensitivity Substrate (Thermo) and ChemiDoc XRS + imaging system (Bio-Rad laboratories). Quantitative analysis of protein bands was performed by the Quantity One 4.62 software.

### 2.12 Statistical analysis

All the experiments were independently repeated at least three times. All data are presented as mean ± SD. Statistical analyses were performed with a two-tailed Student’s t-test by GraphPad Prism 6.0 Software. Differences between groups were considered statistically significant if *p* < 0.05. The statistical significance is denoted by asterisks (*, *p* < 0.05; **, *p* < 0.01; ***, *p* < 0.001).

## 3 Result

### 3.1 Generation of *rpgra* mutant zebrafish using TALENS

Zebrafish *rpgra* genomic sequence (ENSDART00000079095.4) was downloaded from the Ensembl database. The target sites were designed by Internet tools (https://tale-nt.cac.cornell.edu/). In the middle of the TALENs binding sites there is a 17bp spacer containing NsiI restriction enzyme cut site for mutant screening (Figure 1A). Through several rounds of crossing and mutation screening, we obtained a homozygous *rpgra* mutant zebrafish line carrying a deletion-insertion mutation (c.1675_1678delinsTAAGATTGCTTGATGATTGAG) (Figure 1B), which led to the identification of a truncated Rpgra protein p. Met559*. The protein structure of Rpgra^WT^ and Rpgra^p. Met559*^ are displayed in Supplementary Figure S1. Real-time PCR analysis showed that *rpgra* mRNA expression was decreased by 50% in homozygous mutant zebrafish eyes at 2mpf (month post-fertilization) and 6mpf (Figure 1C). Considering the existence of *rpgrb*, the paralogous gene of *rpgra*, we amplified the CDS sequences of two *rpgrb* transcripts using the mutant zebrafish eyes cDNA as a template; the sequences were verified by sequencing, and the results showed that the CDS sequences of the two transcripts of *rpgrb* did not mutate (results not shown). Then in the Western blotting analysis of zebrafish eyes lysate, using an anti-RPGR antibody (Shu et al., 2010), the result showed that Rpgra protein was markedly decreased in mutant lines (Figure 1D); moreover, the levels of the two isoform proteins of Rpgrb did not change. These results indicated that the mutant of *rpgra* is effective. In the rest of the research, we considered the homozygous zebrafish as *rpgra* ^*−/−*^.

### 3.2 *rpgra* ^*−/−*^ zebrafish showed a diminished light response in early development

**FIGURE 2:**
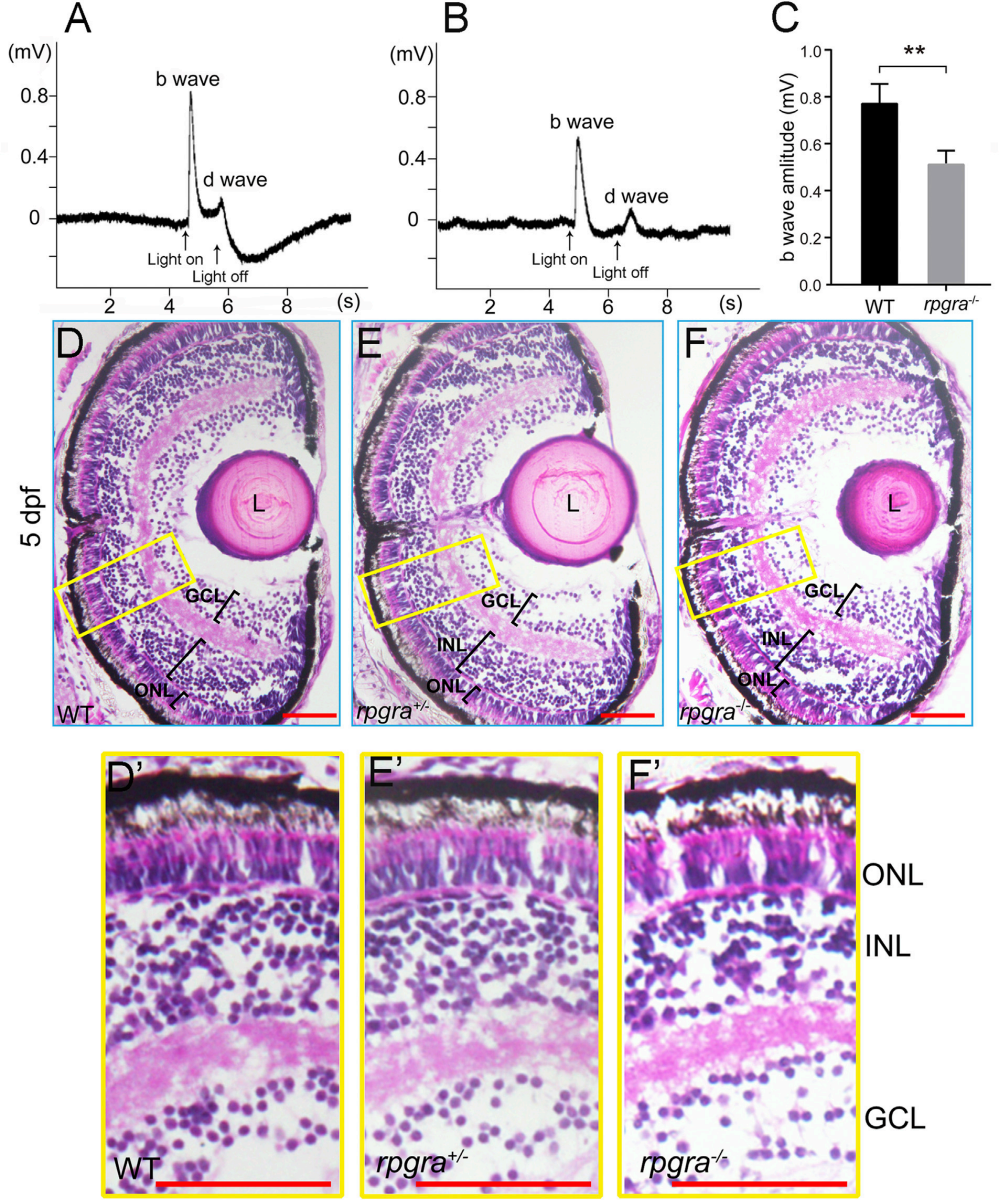
Visual impairment in the *rpgra* ^*−/−*^ larval zebrafish. **(A)** Detection of visual function by ERG analysis of WT zebrafish at 5 dpf. **(B)** Detection of visual function by ERG analysis of *rpgra* ^*−/−*^ zebrafish at 5 dpf. **(C)** Comparison of b-wave amplitudes between WT (n = 5) and *rpgra* ^*−/−*^ (n = 6) zebrafish using two-tailed Student’s t-test. The result is shown as mean ± SD. **, *p* < 0.01. **(D–F)** Histological analyses of WT, *rpgra* ^*+/−*^, and *rpgra* ^*−/−*^ zebrafish at 5dpf. Cryosections were stained with hematoxylin and eosin. **(D′–F′)** Enlarged images of the area inside the yellow box in the **(D–F)**. ONL, outer nuclear layer; INL, inner nuclear layer; GCL, ganglion cell layer; L, Lens. Scale bars, 50 µm.

As reported previously, the loss of RPGR function in human and mouse models causes retinal degeneration with the mislocalization of rod and cone opsin, the reduction of ERG function at early ages, and the progressive loss of photoreceptor cells with aging (Huang et al., 2012; Thompson et al., 2012). In zebrafish, knockdown *rpgrb* and *rpgra* showed different phenotypes. To confirm the role of *rpgra* in the zebrafish retina, we carried out electroretinography (ERG) measurements to check the visual function of mutant zebrafish. The scotopic b-wave amplitudes of *rpgra* ^*−/−*^ zebrafish were significantly decreased compared to the wild-type controls at 5dpf (day post-fertilization) (Figures 2A–C), suggesting that Rpgra deficiency may impact early age visual function.

Then, we conducted a histological analysis using hematoxylin and eosin (H&E) staining of 5dpf zebrafish retina cryosections. Compared with wildtype controls, the retinal lamination displayed no evident difference in heterozygous and homozygous mutant zebrafish (Figures 2D–F, D′–F′). Further, we labeled the outer segments of rods and all four types of cones using antibodies against their respective opsins (Rhodopsin, Opn1lw1, Opn1mw1, Opn1sw2, and Opn1sw1) to identify the rod and cone photoreceptors more specifically. The *rpgra* ^*−/−*^ zebrafish showed no abnormalities in the development of the morphology of photoreceptor cells compared with the wild type at 5dpf (Supplementary Figure S2), suggesting that deficiency of Rpgra did not affect the development of the zebrafish retinal tissue structure.

### 3.3 *rpgra* ^*−/−*^ zebrafish showed progressive retinal degeneration

**FIGURE 3:**
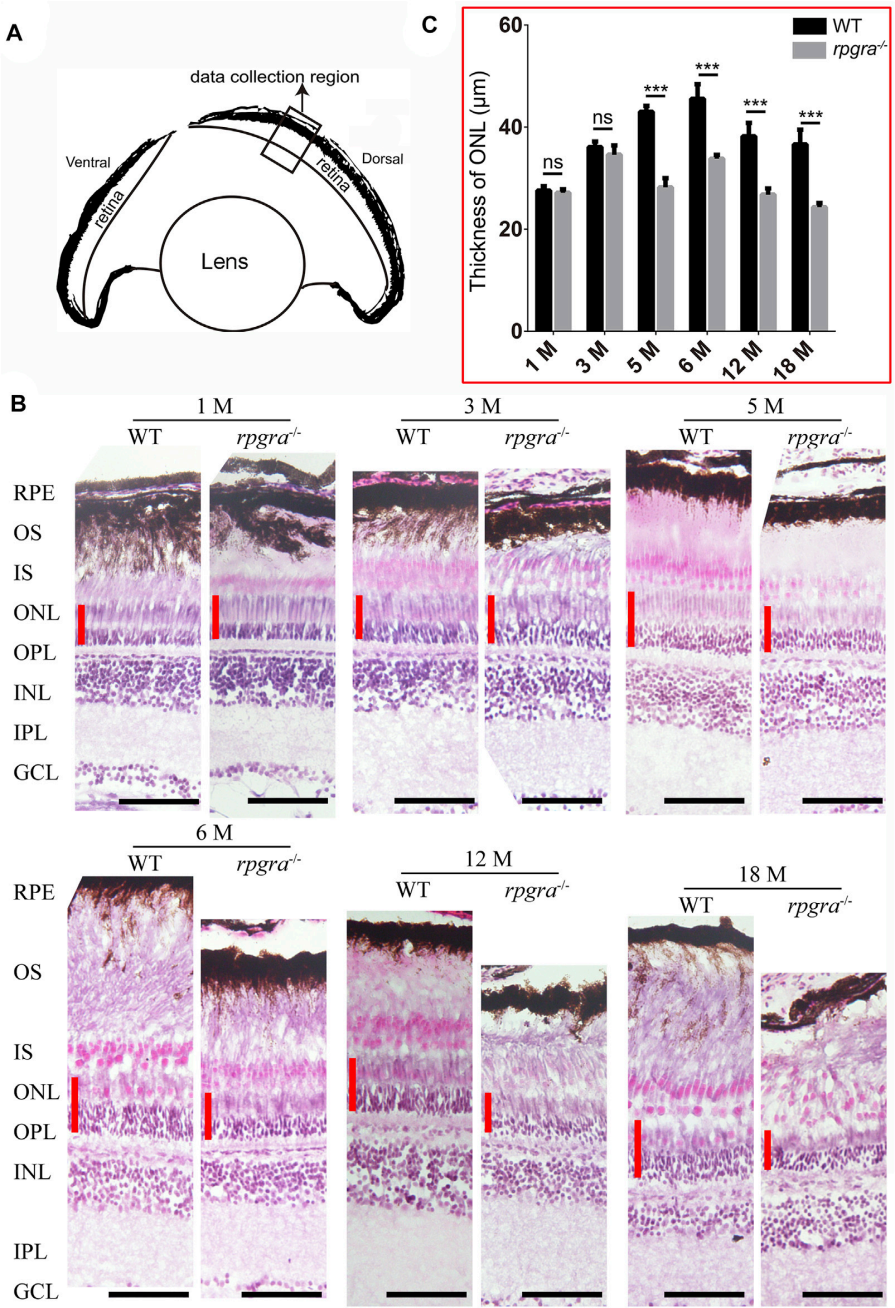
Retinal degeneration in *rpgra* ^*−/−*^ zebrafish revealed by histologic analysis. **(A)** Schematic showing the data collected region of WT or *rpgra* ^*−/−*^ retina. **(B)** Retinal sections from the dark-adapted WT and *rpgra* ^*−/−*^ zebrafish stained with hematoxylin and eosin (H&E). The red lines indicate the thicknesses of the ONL. RPE, retinal pigment epithelium; OS, outer segment; IS, inner segment; ONL, outer nuclear layer; OPL, outer plexiform layer; INL, inner nuclear layer; IPL, inner plexiform layer; GCL, ganglion cell layer. Scale bars, 50 μm. **(C)** Statistical results of the thickness of the retinal outer nuclear layer in each month’s zebrafish (n = 8) were analyzed using a two-tailed Student’s t-test and shown as mean ± SD. **, 0.001 < *p* < 0.01; ***, *p* < 0.001.

To further investigate whether the *rpgra* mutant has an effect on the adult zebrafish retina, H&E staining was performed on retinal sections obtained from both wild-type and *rpgra* ^*−/−*^ zebrafish at various time points ranging from 1 to 18 months post-fertilization (mpf) (Figure 3). Data were collected from the middle segment of the dorsal retina (Figure 3A). The results indicated a significant reduction in the thickness of the outer nuclear layer (ONL) in *rpgra* ^*−/−*^ zebrafish, compared to wild-type control after 5mpf (Figures 3B, C), and the outer segments in mutant zebrafish retina showed disorder with age (Figure 3B, lower panel). In addition, we used TdT-mediated Dutp Nick-end Labeling (TUNEL) staining on retinal cryosections to investigate the extent of apoptosis. Cell death signals were detected in the *rpgra* ^*−/−*^ zebrafish retinas but hardly in the control (Supplementary Figure S3). These indicated the presence of retinal degeneration in *rpgra* ^*−/−*^ zebrafish. Meanwhile, H&E staining was performed on the retinal section of 8-month-old *rpgra* ^*+/−*^ zebrafish, revealing no significant alterations in the retinal structure (Supplementary Figure S4).

**FIGURE 4:**
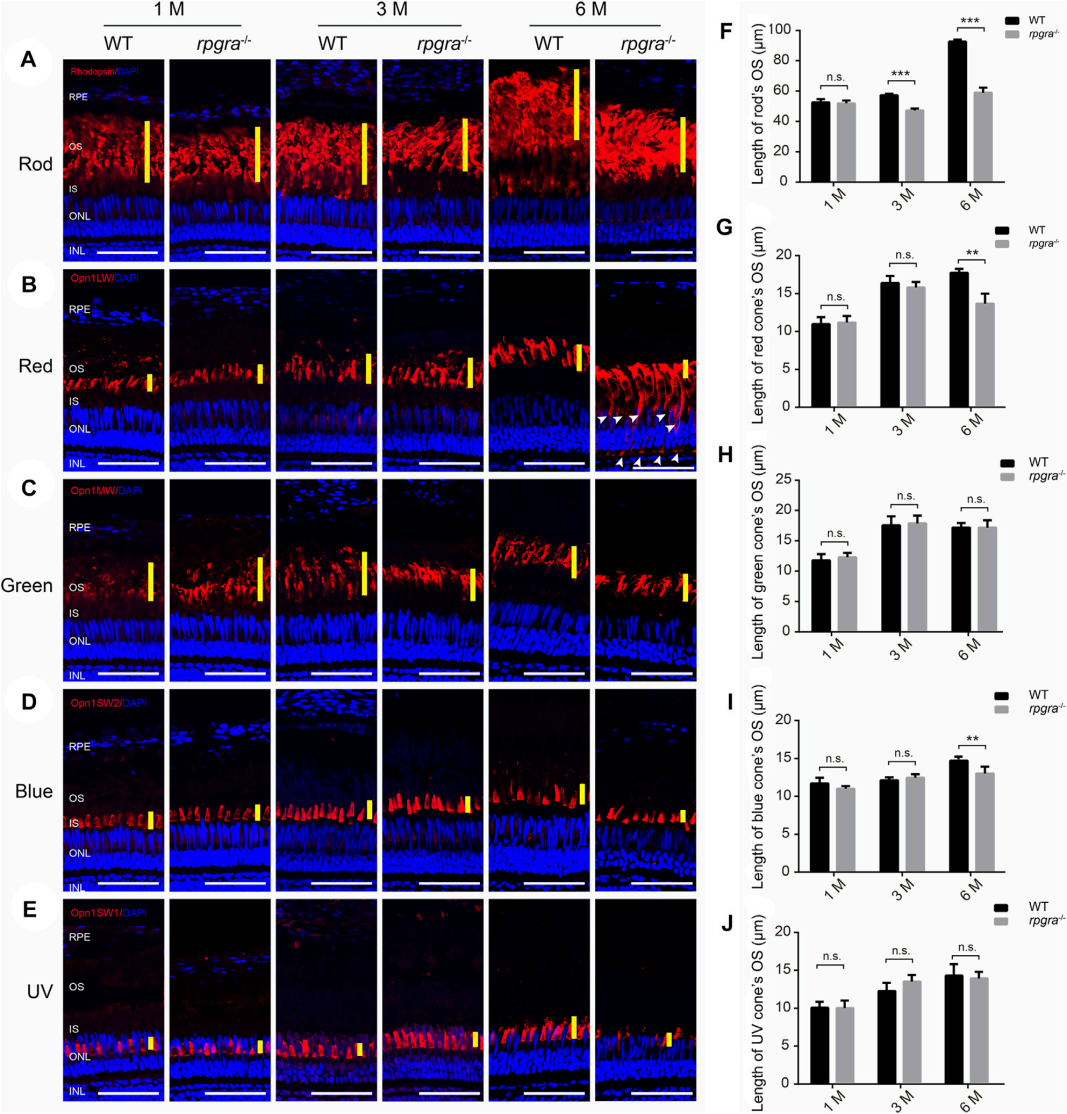
Photoreceptor outer segment is affected in *rpgra* ^*−/−*^ zebrafish. Retinal cryosections from WT and *rpgra* ^*−/−*^ zebrafish were labeled rods **(A)**, red cones **(B)**, green cones **(C)**, blue cones **(D)**, and UV cones **(E)**, with specific antibodies at the ages of 1, 3, and 6 months. The yellow lines indicate the thickness of the outer segment layer of the photoreceptor; the white arrows indicate the mistrafficked Opn1lw1 protein. RPE, retinal pigment epithelium; OS, outer segment; IS, inner segment; ONL, outer nuclear layer; INL, inner nuclear layer. Scale bars, 50 μm. The statistical data are presented in **(F)** for rods and **(G–J)** for cones. At least three images from three eyes of each group were quantified and analyzed using a two-tailed Student’s t-test. The results are shown as mean ± SD. **, *p* < 0.01; ***, *p* < 0.001.

The length of outer segments in mutant zebrafish retina tended to be shorter than that in the wild type (Figure 3B). To confirm this suggestion and distinguish which photoreceptor cell type was affected, we extended our observation by analyzing immunofluorescence of the retinal cryosections, using specific antibodies (rhodopsin, opn1lw, opn1mw, opn1sw2, and opn1sw1) to label the outer segments of the rods and four types of cones (red, green, blue, and UV). Data collection regions were the same as above (Figure 3A). Comparing the changes in the outer segment length of each photoreceptor cell, we found that the outer segments of rod cells in *rpgra* ^*−/−*^ zebrafish became significantly shorter at 3mpf (Figures 4A–F), and for the cone cells, the outer segments of the red and blue cones became shorter at 6mpf, while the green and UV cones did not change significantly until 6mpf (Figures 4B–E, G–J). Similarly, we used specific antibodies to label the rod and red cone outer segments of *rpgra* ^*+/−*^ zebrafish at 8mpf and found no significant changes in the length of the outer segments of the rod and red cone (Supplementary Figures S4B, D–F).

Furthermore, we conducted cone counts at 1, 3, and 6 mpf and observed no significant decrease in cone numbers prior to the age of 6 months (Supplementary Figure S5). We hypothesized that the reduction in thickness of the outer nuclear layer at 6mpf may be attributed to a decline in rod density. To confirm the abnormality of the rod, we first examined the expression of phototransduction proteins through quantitative PCR. The results showed that the expression levels of phototransduction genes in the *rpgra* ^*−/−*^ zebrafish retina were significantly downregulated at 6 mpf compared with the wild type (Supplementary Figure S6), and the protein levels of represented rod-specific genes (Gnat1, Grk1, and Rhodopsin) were significantly decreased in *rpgra* ^*−/−*^ retina, but the protein levels of cone-specific gene (Gnb3 and Gnat2) did not change significantly (Supplementary Figure S6). Taken together, our observations demonstrated that the absence of Rpgra led to progressive retinal degeneration and affected both the rods and cones, with the rods being influenced first.

### 3.4 Abnormal ciliary trafficking in *rpgra* ^*−/−*^ zebrafish retinas

**FIGURE 5:**
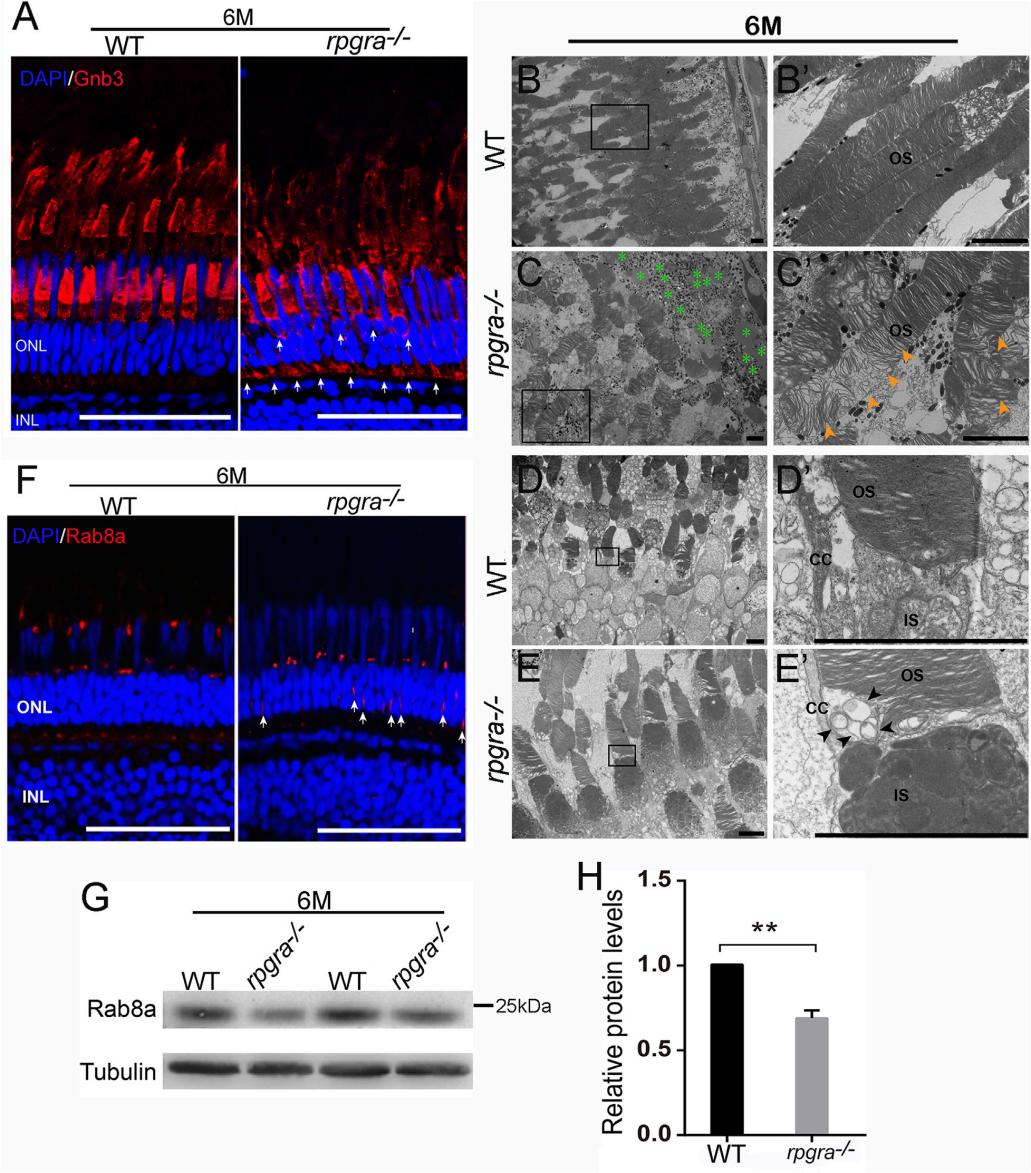
Abnormal ciliary trafficking in *rpgra* ^*−/−*^ zebrafish photoreceptors. **(A)** Retinal cryosections from WT and *rpgra* ^*−/−*^ zebrafish were labeled Gnb3 with specific antibodies at the ages of 6 mpf. Scale bars, 50 μm. **(B)** Well-maintained outer segments (OS) from WT retina. **(B′)** Enlarged image of the box in **(B)**. Scale bars, 5 μm. **(C)** Disorganized and loosely arranged outer segment membrane disc in *rpgra* ^*−/−*^ zebrafish retina. Green asterisks, Lipid droplet. **(C′)** Enlarged image of the box in **(C)**. The yellow arrows show a large number of loosely arranged outer segment membrane discs. Scale bars, 5 μm. **(D)** The complete structure of connecting cilia without the accumulation of abnormal objects in WT photoreceptor cells. **(E)** Obvious accumulation of transport vesicles in the connective cilia of the *rpgra* ^*−/−*^ photoreceptor cells. **(D′, E′)** Enlarged images of the boxes in **(D, E)**. The black arrows indicate the accumulated vesicles. CC, connecting cilia; OS, outer segment; IS, inner segment; Scale bar, 5 μm. **(F)** Immunofluorescence showed that Rab8A protein in 6-month-old *rpgra* ^*−/−*^ zebrafish is mislocated in photoreceptor cells. The arrow marks the mislocated signals; Scale bar, 50 μm. **(G)** Protein levels of Rab8a were detected by Western blot at the age of 6mpf. **(H)** Statistical result of Rab8a protein expression level. The quantitative data of five independent experiments were statistically analyzed using a two-tailed Student’s t-test and shown as mean ± SD. **, *p* < 0.01.

During the above detection, we observed a mislocalization of red opsin in the inner segment, perinuclear space, and outer plexiform layer of 6mpf *rpgra* ^*−/−*^ zebrafish photoreceptors (Figure 4B). To investigate ciliary trafficking of phototransduction components in the *rpgra* ^*−/−*^ zebrafish retina, we detected the protein G-protein beta subunit (Gnb3) which is localized to the outer segments of cone photoreceptors as a marker (Nikonov et al., 2013). Similar mislocalization was observed in the *rpgra* ^*−/−*^ zebrafish retina (Figure 5A). As reported previously, newly formed disk membranes at the base of the photoreceptor outer segments were notably disorganized while the structure of the connecting cilia appeared well maintained in *Rpgr*-KO mice (Hong et al., 2000). To explore the ultrastructural alterations of the photoreceptors in *rpgra* ^*−/−*^ zebrafish, we performed a transmission electron microscopy assay. Compared with wild-type controls, the disk membranes of photoreceptor outer segments exhibited significant disorganization and loose stacking in 6-month-old *rpgra* ^*−/−*^ zebrafish (Figures 5B′, C′). Furthermore, some vesicle-like structures were observed to accumulate around the connecting cilium below the OSs (Figure 5E′).

The small GTPase RAB8A plays a direct role in the trafficking of opsin-carrier vesicles, and defects in RAB8A can result in the accumulation of vesicles within photoreceptors (Moritz et al., 2001; Wang and Deretic, 2014, 2012). Additionally, RPGR^ORF15^ interacts with RAB8A to modulate its intracellular localization and function (Murga-Zamalloa et al., 2010). To investigate whether the expression of Rab8a was affected in *rpgra* deleted zebrafish, we examined Rab8a protein level and its localization using Rab8 antibody. The results showed that Rab8a was mislocalized throughout the cell body in *rpgra* ^*−/−*^ retina, while it was localized to the base of the outer segment in controls (Figure 5F). Subsequently, Western blot results indicated a significant decrease in Rab8a protein levels (Figures 5G, H). Abnormal expression of Rab8a might be one of the reasons for vesicle accumulation and impaired ciliary transport in photoreceptor cells of *rpgra* ^*−/−*^ zebrafish.

## 4 Discussion

Mutations in the *RPGR* gene are associated with X-linked retinitis pigmentosa. Approximately 75% of all XLRP cases are caused by mutations in RPGR ORF15, which is a highly repetitive and purine-rich region, thus considered a hotspot for mutations (Iannaccone et al., 2004; Vervoort et al., 2000). Most ORF15 mutations cause truncation of the C-terminal domain of the RPGR protein, and these mutations are associated with slightly milder disease than mutations in the N-terminal RCC1-like domain (Sharon et al., 2003). Mutations in the RCC1-like domain may impact the interaction of RPGR with other proteins, leading to aberrant cellular function (Megaw et al., 2015). However, the function of the RPGR exon ORF15 repeat domain remains unclear. The phenotypes resulting from *RPGR* mutations display heterogeneity across diverse genetic backgrounds (Huang et al., 2012), necessitating the use of multiple animal models to more comprehensively elucidate the function of ORF15.

In this study, we generated a new *rpgra* mutant model to provide a new perspective for research into the function of *RPGR* ^*ORF15*^. The mutant zebrafish exhibited visual impairment at 5 dpf, while the retinal structure remained normal until 5 mpf, and the length of the rod outer segments was reduced at 3 mpf, accompanied by a slight downregulation of the rhodopsin protein level (data not shown). The thickness of the outer nuclear layer in mutant zebrafish retina progressively decreases, accompanied by shortening and disarrangement of rod outer segments, as well as a reduction in length of the cone’s outer segment at 6mpf. However, the number of cones remained unchanged compared with the wild type, suggesting that the reduction of rods may be the primary cause for the thinning of the outer nuclear layer. Increased apoptotic signaling served as an additional indicator of cellular degeneration in the mutant retina (Chang et al., 1993; Li et al., 2014). These alterations bear a striking resemblance to certain RP models of zebrafish (Liu et al., 2015; Noel et al., 2020; Yu et al., 2017). However, the retinal phenotype of *rpgra* mutant zebrafish is relatively mild compared to some *RPGR* ^*ORF15*^ patients (Huang et al., 2012).

In zebrafish, there are two homologous genes, *rpgra* and *rpgrb*, both of which show high expression patterns in the zebrafish embryonic retina, brain, and neural tube and are expressed at the connecting cilia of adult zebrafish optic photoreceptor cells. Bioinformatic alignments revealed the *rpgra* is homologous to the *RPGR* ^*ORF15*^ in mammals; in contrast, the *rpgrb* has two transcripts that are homologous to *RPGR* ^*ORF15*^ and *RPGR* ^*ex1-19*^, respectively (Shu et al., 2010). Thus, we suspect that the presence of *rpgrb* ^*ORF15*^ may modulate the severity of the *rpgra* mutated zebrafish phenotype. However, protein assays showed that *rpgra* ^*ORF15*^ was highly expressed in adult zebrafish eyes, followed by *rpgrb* ^*ex1-17*^, and *rpgrb* ^*ORF1*5^ was the lowest (Figure 1D). Previous studies showed that knockdown of *rpgrb* ^*ORF15*^ expression in zebrafish resulted in reduced length of Kupffer’s vesicle (KV) cilia and is associated with ciliary anomalies including shortened body-axis, kinked tail, hydrocephaly, and edema but does not affect retinal development (Ghosh et al., 2010). In contrast, the inhibition of *rpgra* expression was not detected as a significant defect (Shu et al., 2010). This suggests that *rpgrb* ^*ORF15*^ plays a more crucial role in the early developmental stages of zebrafish, while *rpgra* exerts dominance in the eyes of adult zebrafish. Moving forward, targeted disruption of *rpgrb* ^*ORF15*^ in zebrafish can be performed and combined with *rpgra* mutant zebrafish to investigate their distinctions and complement the role of *rpgr* ^*ORF15*^ in zebrafish.

Compared to rd9 mice and XLPRA1 dogs, the phenotypic similarities between *rpgra* mutant zebrafish and rd9 mice were more pronounced. These include the presence of retinal pathology and the reduction of ERG function at early ages. Our analysis of the disease progression in *rpgra* mutant zebrafish showed that ERG b-wave amplitudes were reduced as early as 5dpf, consistent with the temporal expression of *rpgra*. Unfortunately, the ERG test we used was only applicable to assess zebrafish larvae, so we could not monitor the subsequent progression of visual loss. Further study of the changes in the inner retina of rd9 mice showed that although the cone-rod was intact, slow or absent renewal of outer segments may affect the synaptic level, resulting in a worsening of the transfer of information from photoreceptors to inner retinal neurons. It demonstrated that alterations in retinal physiology can be detected before any major morphological change besides rod loss (Falasconi et al., 2019).

In addition to detecting the abnormal outer nuclear layer and outer segment, we also found that the red cone opsin (opn1lw1) and G-protein beta subunit (Gnb3) were mislocalized to the IS and INL in *rpgra* ^*−/−*^ zebrafish retina at 6 months, while Rhodopsin did not appear to be mislocalized (Figures 4B, 5A). This is similar to the phenotype of rd9 mice (Thompson et al., 2012; Zhang et al., 2019), suggesting that zebrafish *rpgra* may be functionally homologous to the mouse *Rpgr* ^*ORF15*^, and the *rpgra* mutant zebrafish model constructed in this experiment is suitable for the functional and pathological studies of *RPGR* ^*ORF15*^.

Some other retina degeneration animal models such as *CC2D2A*, *RP2*, and *EYS* are characterized by opsin mislocalization and further photoreceptor cell loss, and these genes are associated with cilia transport (Liu et al., 2015; Moritz et al., 2001; Renault et al., 2001). The specific function of RPGR in photoreceptor cell ciliary transport has been determined (Bachmann-Gagescu et al., 2011; Deretic et al., 1995; Moritz et al., 2001; Renault et al., 2001). RPGR^ORF15^ is localized to the connecting cilium of the photoreceptors and may be involved as cargo in protein transport processes (Khanna et al., 2005). Given this, we used electron transmission microscopy to observe changes in the subcellular structure of photoreceptor cell membrane discs and connecting cilia; the result showed that the membrane discs of the *rpgra* ^*−/−*^ zebrafish photoreceptor cells were loosely disorganized, and some vesicle-like structures were accumulated around the connecting cilium below the OSs, showing a resemblance phenotype occurring in *cc2d2a* and *Whirlin* defect models (Bachmann-Gagescu et al., 2011; Yang et al., 2010). Combined with the observed unaffected internal structure of the connecting cilia in *Rpgr*-ko mouse photoreceptor cells (Hong et al., 2000), we considered that *rpgra* deletion mainly affects the protein transport process in connecting cilia.

RPGR is a GTPase regulator whose domain encoded by exons 1–11 is homologous to chromosome condensation regulator 1 (RCC1), which is a small GTPase guanine exchange factor (GEF). GEFs can catalyze the conversion of inactive GDP-bound GTPases to the active GTP-bound form (Renault et al., 2001). The application of different N-terminal regions of RPGR revealed that RPGR preferentially interacts with the GDP-bound form of the GTPase RAB8A and catalyzes the conversion of RAB8A-GDP to RAB8A-GTP. Knockdown of RPGR expression in hTERT-RPE1 cells resulted in reduced retention of RAB8A at the cilia and shortened cilia length (Murga-Zamalloa et al., 2010). In the present study, a significant downregulation and mislocalization of Rab8a protein showed in *rpgra* ^*−/−*^ zebrafish eyes (Figures 5F–H). RAB8A is a major participant in rhodopsin-bearing vesicle trafficking and plays a critical role in the delivery of rhodopsin-containing post-Golgi vesicles to the base of the connecting cilium (Deretic et al., 1995; Moritz et al., 2001). Furthermore, the MICAL3-NINL-CC2D2A complex interacting with RAB8A is required for correct opsin-carrier-vesicle fusion at the periciliary membrane (Bachmann-Gagescu et al., 2015; Ojeda et al., 2017). However, there was no mislocalization of rhodopsin in *rpgra* ^*−/−*^ zebrafish eyes (Figure 4A). Based on these, we hypothesize that in the zebrafish retina, Rpgra or Rab8a is involved in cones and rods protein transport through different mechanisms. Further investigation is needed to determine whether the protein specificity of Rab8a-related vesicle trafficking is directly regulated by Rpgra.

In addition, we found a large accumulation of lipid droplets in the retinal pigment epithelium (RPE) layer of the *rpgra* ^*−/−*^ zebrafish retina (Figure 5C). Although there have been no reports of lipid accumulation in the retinal pigment epithelium (RPE) in animal models or patients with RPGR mutations, macular degeneration has been observed in some *RPGR* ^*ORF15*^ patients, and altered RPE integrity has also been noted in rd9 mice (Charng et al., 2016; Falasconi et al., 2019). These findings suggest that RPGR may have an important role in RPE. More importantly, lipid droplets were already present in zebrafish RPE at 3 months, which is consistent with the time of the rod outer segment degeneration (data not shown). Further research is needed to determine whether the abnormality of RPE in *rpgra* ^*−/−*^ zebrafish is associated with photoreceptor cell degeneration, especially the degeneration of the rod’s outer segment. Also, the function of RPGR^ORF15^ in RPE is worthy of further exploration.

## 5 Conclusion

In conclusion, we have successfully established a novel *rpgra* mutant zebrafish model that exhibits a retinal degenerative phenotype. Our findings confirm the essential role of Rpgra in opsin protein transport from inner segments through the connecting cilium to outer segments in the zebrafish retina. This model provides an opportunity for future investigation into the cellular function of *RPGR* ^*ORF15*^ and elucidation of the underlying disease mechanisms, as well as enabling the development of drug candidates for the treatment of conditions caused by *RPGR* ^*ORF15*^ mutations.

## Data Availability

The original contributions presented in the study are included in the article/Supplementary Material; further inquiries can be directed to the corresponding author.
