# Supplementary material for: Retinal degeneration in rpgra mutant zebrafish
Source: Front Cell Dev Biol. 2023 Jun 7;11:1169941. doi: 10.3389/fcell.2023.1169941 (PMC10282147; doi:10.3389/fcell.2023.1169941)
Supplement: Supplementary file 1 [file DataSheet1.docx]

Supplementary Material

**Retinal degeneration in *rpgra* mutant zebrafish**

**Xiliang Liu ^1,^****^6†^, Shanshan Han ^2†^, Fei Liu ^1,3^, Shanshan Yu ^1,4^, Yayun Qin ^1,5^, Jingzhen Li ^1^,** **Danna Jia ^1^, Pan Gao ^1^, Xiang Chen ^1^, Zhaohui Tang ^1^, Mugen Liu^1^, Yuwen Huang ^1,^***

^+^Xiliang Liu and Shanshan Han contributed equally to this work.

*** Correspondence:**Yuwen Huang

[ywhuang1994@hust.edu.cn](mailto:ywhuang1994@hust.edu.cn)

# Supplementary Tables

**Table S1.** List of primary antibodies used in this paper

| **Antibodies** | **Source** | **Dilution** |
| --- | --- | --- |
| Anti-α-Tubulin | Millipore, FCMAB322PE | 1:3000 for WB |
| Anti-GAPDH | Proteintech, 10494–1-AP | 1:3000 for WB |
| Anti-RPGR | Sigma, HPA001593 | 1:1000 for WB |
| Anti-Rhodopsin | Abclonal, Customized | 1:100 for IF |
| Anti- Opn1lw | Abclonal, Customized | 1:100 for IF |
| Anti-Opn1mw | Abclonal, Customized | 1:100 for IF |
| Anti-Opn1sw1 | Abclonal, Customized | 1:100 for IF |
| Anti-Opn1sw2 | Abcepta, Q9W6A8 | 1:50 for IF |
| Anti-GNB3 | Proteintech, 10081-1-AP | 1:200 for IF |
| Anti-RAB8a | Abcam, ab188574 | 1:100 for IF; 1:1000 for WB |

**Table S2.** Primers used for RT-PCR

| **Primers** | **Sequences (5’->3’)** |
| --- | --- |
| *gnb1a-*F | agatcagagatgcgcggaaag |
| *gnb1a-*R | caagtgtcccctcagtgtcc |
| *gnb1b-*F | actatcacagatcacagccaaca |
| *gnb1b-*R | gtgcatggcgtagattttagc |
| *gnb3a-*F | acgccattgggtttttcccca |
| *gnb3a-*R | ggacgtcacgccgcacatga |
| *gnb3b-*F | cacagattgaggcggctcgca |
| *gnb3b-*R | agttggacccgaggggctgg |
| *gnat1-*F | acgtcctgcgttcaagagtg |
| *gnat1-*R | atgtggtggcgaagtaacgg |
| *gnat2-*F | tcgccatctgcacaggaggat |
| *gnat2-*R | gctctggaggcatggtgccc |
| *gnat2-*R | gctctggaggcatggtgccc |
| *recoverin-*F | attcccaaagaggaccaaga |
| *recoverin-*R | tcagccaatcgctcgttat |
| *guca1a-*F | cgacatcaatggggatgggg |
| *guca1a-*R | atggccacgatgtgtgtgag |
| *pde6a-*F | cagtcaacaagatcggggct |
| *pde6a-*R | agctcaggtgaaacactcgg |
| *pde6b-*F | ggagcagccaccttactctg |
| *pde6b-*R | gccaaagcccaatgatctgc |
| *pde6c-*F | acggtgcgtaagggctac |
| *pde6c-*R | gatgcgctctttgtctggta |
| *grk7a-*F | gcttatgacaccaagacccac |
| *grk7a-*R | cgatccatttcgattccct |
| *grk7b-*F | tgcaggttgtcagaccttgg |
| *grk7b-*R | accagtccactgaggtacga |
| *rpgra-*F | tctcctcacctcttccaacct |
| *rpgra-*R | gtaggatggtgcctgttaggg |
| *acb1b-*F | cgagcaggagatgggaacc |
| *acb1b-*R | caacggaaacgctcattgc |

# Supplementary Figures

**
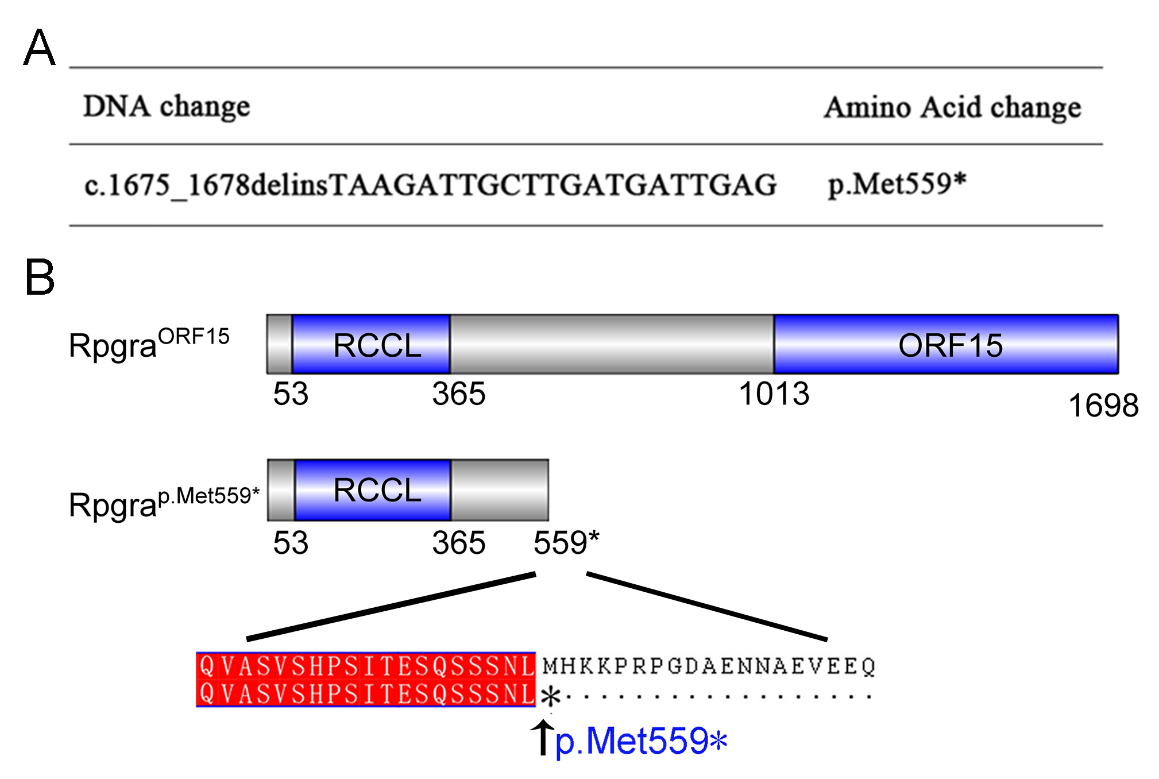
**

**Supplementary Figure 1**. Deletion-insertion of *rpgra* gene generated a truncated protein. (A) Information on DNA and protein changes in *rpgra^-/-^* zebrafish. (B) Schematic diagram of Rpgra^WT^ and Rpgra^p.Met559*^ protein.


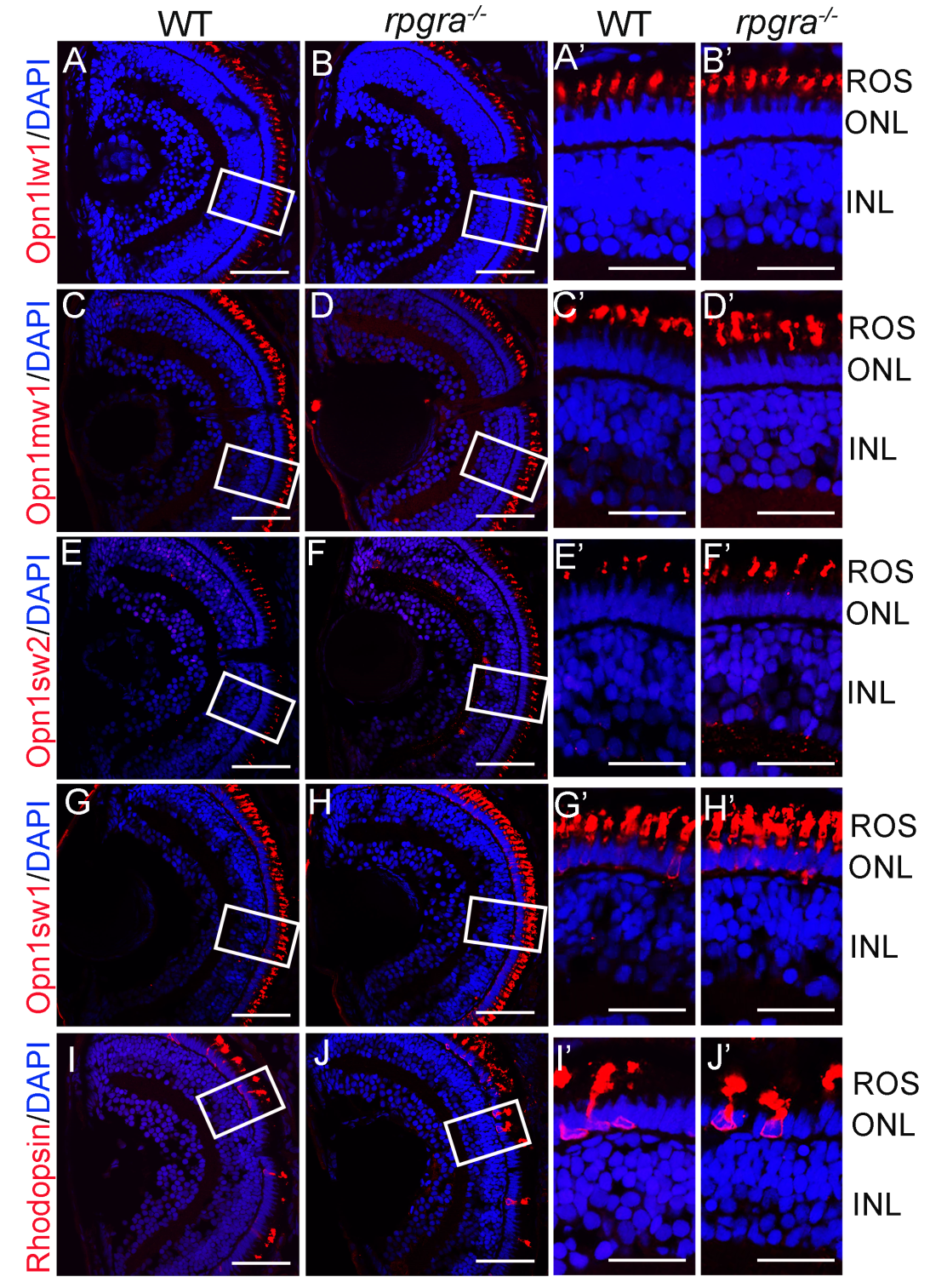


**Supplementary Figure 2**. Rpgra is not essential for the early development of zebrafish retina. Retinal cryosections from WT and *rpgra^-/-^* zebrafish were labelled red cones (A, B), green cones (C, D), blue cones (E,F), UV cones (G, H) and rods (I,J) with specific antibodies at 5dpf. High magnification images of the box in (A- J) was showed in (A’- J’) correspondingly. ROS, retina outer segment; ONL, outer nuclear layer; INL, inner nuclear layer. Scale bar, 50 μm.


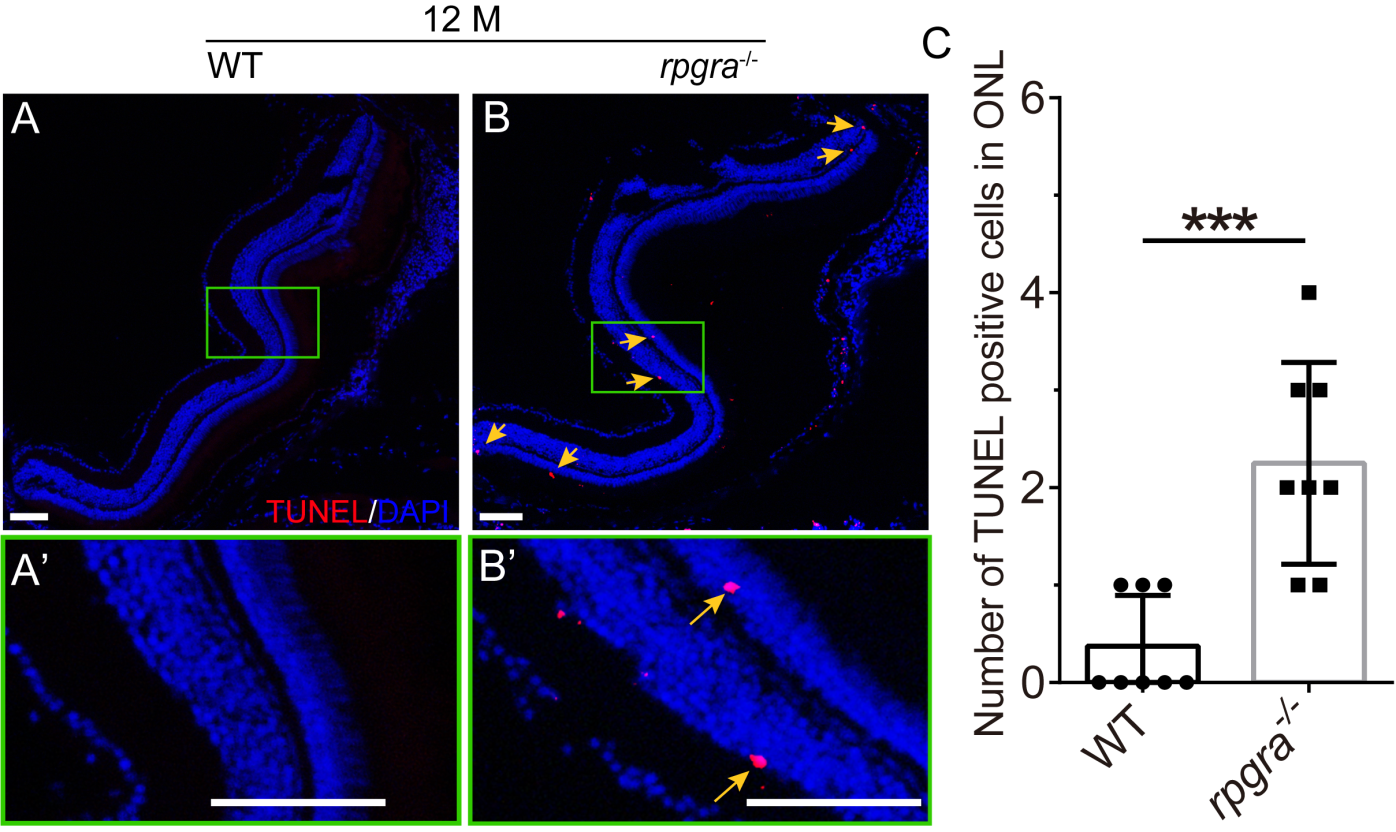


**Supplementary Figure 3**. The degeneration of photoreceptors in *rpgra^-/-^* zebrafish retina was caused by cell death. (A, B) TUNEL staining of WT and *rpgra^-/-^* zebrafish at 12 mpf. Yellow arrows indicated the TUNEL-positive signals (red). (A’, B’) High magnification images of the boxes in (A, B). Scale bars, 100 μm. (C) Quantification of TUNEL positive cells in outer nuclear layers of WT and *rpgra^-/-^* zebrafish at 12mpf (n=8). The results are shown as mean ± SD. **, *p*< 0.01.


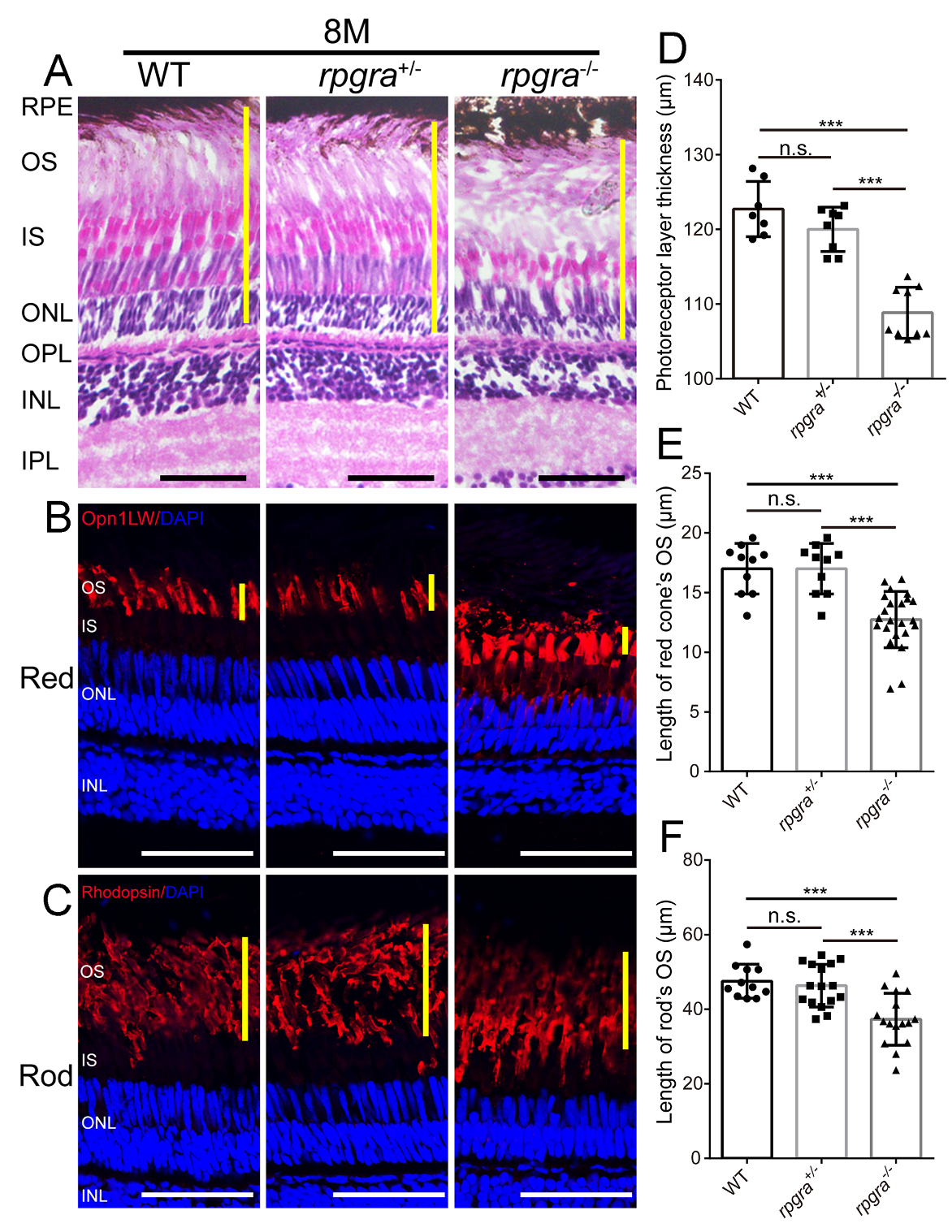


**Supplementary Figure 4.** No significant phenotypic alterations in the retina of heterozygous *rpgra^+/-^* zebrafish. (A) Retinal sections from the dark-adapted WT, *rpgra^+/-^* and *rpgra^-/-^* zebrafish at 8mpf stained with hematoxylin and eosin (H&E). The yellow lines indicate the thicknesses of the photoreceptor layers (outer retina). Retinal cryosections from WT, *rpgra^+/-^* and *rpgra^-/-^* zebrafish were labelled red cones (B), rods (C) with specific antibodies at the ages of 8 months. The yellow lines indicated the thickness of outer segment layer of photoreceptor. RPE, retinal pigment epithelium; OS, outer segment; IS, inner segment; ONL, outer nuclear layer; OPL, outer plexiform layer; INL, inner nuclear layer; IPL, inner plexiform layer. Scale bars, 50μm. (D) Statistical results of the thickness of the retinal photoreceptor layers in (A). (E) Statistical results of the thickness of the length of outer segment of red cones in (B). (F) Statistical results of the thickness of the length of outer segment of rods in (C). Analyzed using two-tailed Student’s t-test and shown as mean ± SD. ***, p<0.001.


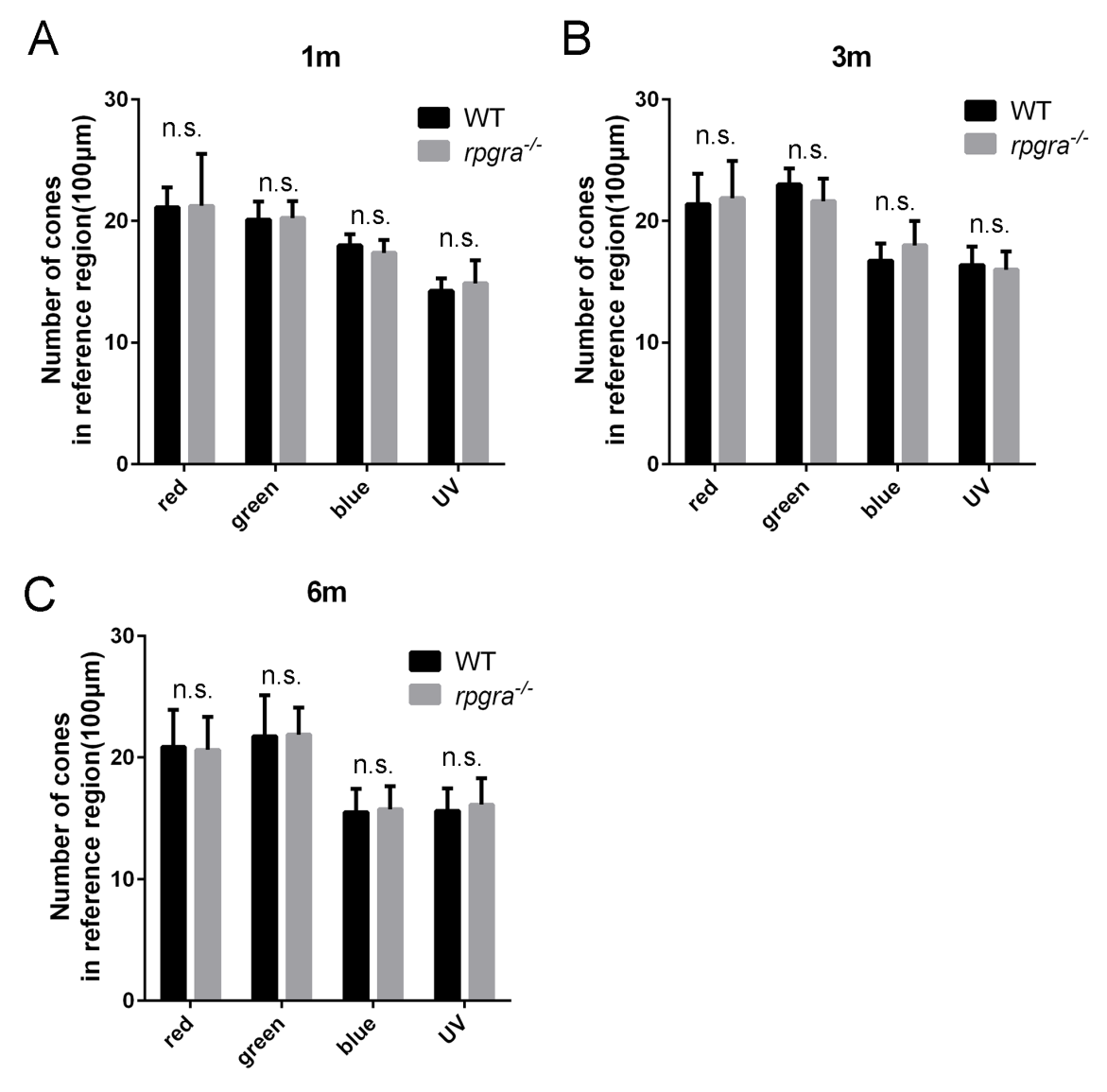


**Supplementary Figure 5.** Statistical data for cones at indicated ages. (A) Quantification of cones in reference region (100μm) of WT and *rpgra^-/-^* zebrafish retina sections at 1mpf. (B) Quantification of cones in reference region (100μm) of WT and *rpgra^-/-^* zebrafish retina sections at 3mpf. (C) Quantification of cones in reference region (100μm) of WT and *rpgra^-/-^* zebrafish retina sections at 6mpf. The data are shown as mean with SD.


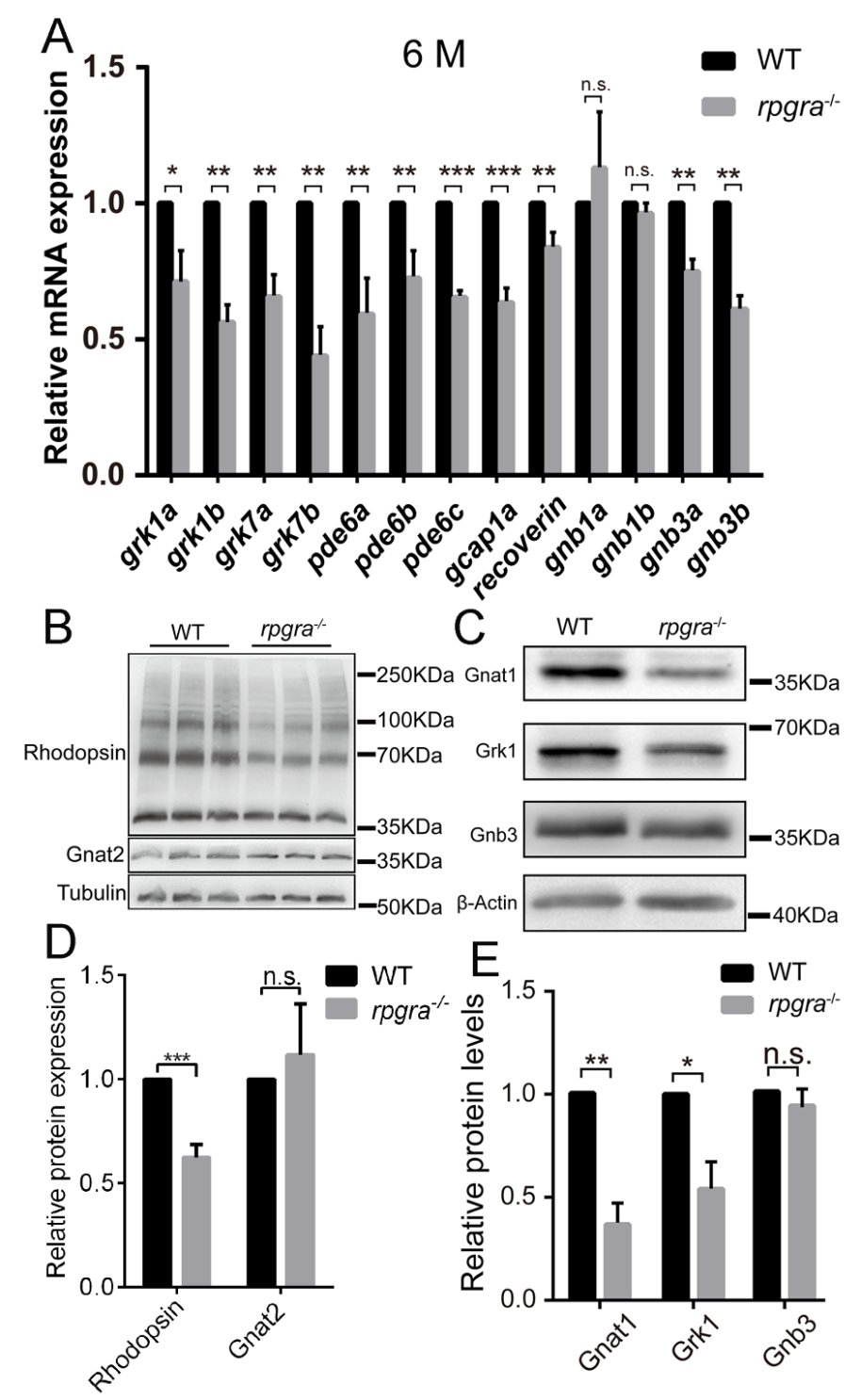


**Supplementary Figure 6**. The expression of most phototransduction proteins was decreased in *rpgra^-/-^* zebrafish retina. (A) The mRNA levels of phototransduction related genes were detected by fluorescence quantitative PCR in WT and *rpgra^-/-^* zebrafish eyes at 6 months of age. Actb1 was served as endogenous control. The data of three independent experiments were analyzed using two-tailed Student’s t-test and shown as mean ± SD. *, *p*<0.05; **, *p*< 0.01; ***, *p*<0.001. (B) The protein levels of Rhodopsin and Gnat2 in WT and *rpgra^-/-^* retinas from 5mpf were evaluated using western blotting. Tubulin was used as a loading control. (C) The protein levels of rod-specific (Gnat1 and Grk1) and cone-specific (Gnb3) genes in WT and *rpgra^-/-^* retinas from 6mpf were evaluated using western blotting. Actin was used as a loading control. (D&E) Quantitative analysis of the protein levels of rod- and cone-specific genes based on at least three independent experiments. The data are shown as mean with SD. *, *p*<0.05; **, *p*<0.01, ***, *p*<0.001.
